# Disentangling the paradox of inbreeding and telomere dynamics

**DOI:** 10.1098/rstb.2026.0110

**Published:** 2026-11-12

**Authors:** Michael L. Pepke, Morten T. Limborg

**Affiliations:** Center for Evolutionary Hologenomics, Globe Institute, Faculty of Health and Medical Sciences, University of Copenhagen, Copenhagen, Denmark

**Keywords:** ageing, biomarker, captivity, conservation, domestication, hybridization, inbreeding depression, life-history, stress, telomere length

## Abstract

Inbreeding can have both profound and subtle fitness consequences, yet the physiological and molecular mechanisms underlying inbreeding depression remain difficult to study in the wild. Telomeres are repetitive DNA sequences protecting chromosome ends, and telomere length (TL) changes are widely used biomarkers of health, ageing and fitness in animal populations. However, studies have reported inconsistent associations between TL and inbreeding, including negative, positive or null relationships. Here we synthesize evidence across eukaryotes and reveal a contrasting pattern. When comparing species or strains with assumed different histories of inbreeding owing to domestication or captive breeding, most studies inferred positive associations between TL and inbreeding. However, when comparing TL with measured individual levels of inbreeding within natural populations, including humans, most studies found negative associations between TL and inbreeding. Within both comparison types, there is very little evidence to evaluate the magnitude and reliability of the influences of domestication and inbreeding, respectively, on TL. We discuss how the longer telomeres of domesticated and laboratory organisms may reflect correlated effects of captive breeding and artificial selection rather than inbreeding. Furthermore, if inbreeding shortens telomeres in natural populations, this has implications for interpreting telomere biomarkers in evolutionary and conservation contexts.

This article is part of the theme issue ‘Developmental circuits in ageing’.

## 1. Introduction

### (a) The paradox of inbreeding effects on telomere dynamics

Inbreeding—mating among related individuals—reduces heterozygosity and can expose deleterious recessive alleles, leading to inbreeding depression in fitness-related traits such as growth, fertility and survival [1,2]. Combined, inbreeding can be a powerful modulator of developmental processes, potentially shaping individual ageing trajectories from early life [3]. Inbreeding depression is a universal phenomenon in diploids, but the genetic underpinnings of inbreeding depression vary among species and populations, and the physiological mechanisms underlying inbreeding depression are poorly understood [2,4]. Identifying biomarkers that link genetic architecture with organismal condition is therefore a key challenge for evolutionary biology, conservation genetics and ecological physiology [5].

Telomeres, the repetitive nucleoprotein complexes capping chromosome ends, play a central role in preserving genome integrity and regulating cellular lifespan [6]. Maintaining functional telomeres is required for the maintenance of eukaryotic life, and their progressive shortening with each cell division and oxidative stress has been widely implicated in ageing, fitness variation and disease susceptibility in different species [7–10]. Telomere length (TL) is therefore considered both an integrative biomarker of physiological state and a potential mediator of life-history trade-offs [5].

Inbred individuals have been shown to have higher metabolism and higher oxidative stress levels [11–14] and reduced immune responses [15]. Higher levels of inbreeding would therefore be expected to be associated with shorter telomeres, but several studies have recently pointed out discrepant results on the associations between telomere dynamics (i.e. TL and/or the change in TL) and inbreeding [7,16–18], inferring both negative associations [17,19,20], positive associations [21–26] or no associations [16,27]. This suggests that further research across taxa and environmental contexts is needed to clarify how inbreeding influences TL and maintenance.

We therefore synthesized evidence of associations between telomere dynamics and inbreeding across four decades of studies on eukaryotes. Most of these studies either compare telomere dynamics with various individual measures of realized or predicted inbreeding or they compare telomere dynamics across populations and strains with assumed different levels of inbreeding owing to domestication or breeding in captivity, which generally increase inbreeding [28]. Understanding how inbreeding influences telomere dynamics is therefore important to how we interpret telomere biomarkers in evolutionary and conservation contexts [9], but also to unravel the factors shaping variation in telomere dynamics across and within species.

### (b) Literature search

We performed a systematic literature search using the *Web of Science* database (WoS, Clarivate; date of last search: April 6 2026 [29]) for titles, abstracts or keywords of peer-reviewed articles (excluding reviews, opinions, perspectives etc.) in English using the search string: (‘telomere length’ OR ‘telomere dynamics’ OR ‘telomere shortening’ OR ‘telomere attrition’ OR ‘telomere lengthening’ OR ‘telomere elongation’ OR ‘telomere maintenance’ OR ‘telomere loss’ OR ‘telomerase’) AND (‘inbre*’ OR ‘outbre*’ OR ‘heterozygosity’ OR ‘homozygosity’ OR ‘consanguin*’ OR ‘heterosis’ OR ‘domestic*’). In addition, we performed forward and backward reference searches of the identified publications for studies related to the topic using WoS and Google Scholar. All studies were individually screened to assess whether they compared TLs, changes in TLs or telomerase activity between individuals or groups with either measured or assumed different levels of inbreeding in eukaryotic organisms. Inbreeding measures therefore include individual estimates of, e.g. heterozygosity and inbreeding coefficients, or a comparison between inbred or domesticated and outbred or wild species or strains. In addition, we assessed whether the studies were conducted in captive or natural environments and whether the studies included domesticated species. Although telomere dynamics often vary across tissues, TLs are generally correlated across tissues within vertebrates (e.g. [30]). TLs were measured using different methods across studies, but the direction of effects is largely congruent across methodologies [31]. We therefore included studies using different methods to measure TL as well as in different tissues (supplementary material, table S1). The PRISMA diagram in figure S1 in the supplementary material shows an overview of the literature search and filtering process.

### (c) Data synthesis

Owing to the large heterogeneity in study designs, sample sizes and methodologies, as well as modes of inference, including correlations, regressions, comparisons of means or individuals, incomplete reporting and testing across different taxonomic scales and different measures of inbreeding, we initially performed a qualitative vote-counting procedure [32] based on the direction of reported effects of inbreeding on TL (i.e. ‘positive’, ‘negative’ or ‘none’). To test whether the direction of associations differed between comparison types, i.e. within natural populations or across groups with assumed different histories of inbreeding owing to, for example, domestication or captive breeding (table 1), we performed Fisher's exact test on studies reporting positive versus negative associations, thus excluding null results with a significance threshold of *p *< 0.05. The vote counting method is a poor approach for assessing the reliability or magnitude of an effect because it assumes that each investigation provides an independent test that has equal value [32]. However, it is valuable in this case where there is controversy regarding the overall direction of an effect across a limited number of very heterogeneous studies (e.g. [62]).

**Table 1.** Overview of 37 studies on the association between telomere length (TL), the change in TL or telomerase activity and inbreeding, domestication or hybridization in eukaryotes, separated into studies that (A) compare different species or strains with assumed different histories of inbreeding owing to domestication or a long history of captive breeding and (B) compare TL with measured levels of inbreeding within natural or wild populations, including humans. The table includes the overall inferred direction of the association between TL and inbreeding level, referring to the summary of the main results and measure of inbreeding, sample size (number of individuals or strains), whether the study was carried out in captivity or wild (natural) environments and whether it included species with a long history of human domestication. If associations are reported for both the total dataset and a data subset, the subset is reported in parentheses. PARN: poly(A)‐specific ribonuclease; RTEL1: regulator of telomere elongation helicase 1; SNP: single nucleotide polymorphism; TERT: telomerase reverse transcriptase.

species	main results	TL-inbreeding direction	sample size	captive or wild?	domesticated?	inbreeding measure	references
A (22 studies)							
maize (*Zea mays*)	crossing recombinant inbred lines of maize resulted in F1 hybrids that had TLs similar to one of the two parental lines.	none	22 inbred lines and four hybrid lines.	captive	yes	comparing strains/species	[33]*
pearl millet (*Pennisetum* spp. [*Cenchrus* spp.])	inbred cultivated lines of *P. glaucum* (*C. americanus*) generally had longer telomeres (20–42 kb) than wild *Pennisetum* species (10–22 kb, *p* = 0.036).	positive	14 strains across seven species	captive	yes	comparing strains/species	[34]*
thale cress (*Arabidopsis thaliana*)	inter-ecotype F1 hybrids TL intermediate to that of the parents.	none	16 crosses	captive	yes	comparing strains/species	[35]
yeast (*Saccharomyces cerevisiae*)	telomeres in a wild strain were shorter than in a laboratory strain. Hybrids of these strains had intermediate TL.	positive	two strains	captive	yes	comparing strains/species	[36]*
yeast (*Saccharomyces cerevisiae* and *S. paradoxus*)	telomeres of domesticated *S. cerevisiae* strains were longer than in wild isolates. Isolates derived from fermentation-related processes had longer TL than feral strains having gone through domestication but redispersed into a natural environment. Domesticated *S. cerevisiae* had longer TL than its closest related species, *S. paradoxus*, which was never domesticated.	positive	706 isolates	captive	yes	comparing strains/species	[21]*
yeast (*Saccharomyces cerevisiae*)	TL was not significantly longer in domesticated versus wild strains (*p* = 0.17) or in heterozygous versus homozygous strains (*p* = 0.21).	none	142 strains	captive	yes	comparing strains/species	[37]*
nematode (*Caenorhabditis elegans*)	a laboratory strain had longer TL (17 kb) than most wild strains (median 12 kb), but wild strains ranged from 4–83 kb.	positive (none)	208 strains (152 isotypes)	captive	yes^1^	comparing strains/species	[38]
turquoise killifish (*Nothobranchius furzeri*)	a recently wild-derived strain showed age-dependent telomere shortening despite increased telomerase activity, but there was no change in TL or telomerase activity in an inbred laboratory strain.	positive	two strains	captive	no^1^	comparing strains/species	[39]
killifish (*Nothobranchius* spp.)	an inbred laboratory strain of turquoise killifish, *N. furzeri,* had longer TL compared with a more recently wild-derived *N. furzeri* strain and five other wild *Nothobranchius* species.	positive	nine strains across six species	captive	no^1^	comparing strains/species	[26]*
chicken (*Gallus gallus*)	two domestic chicken breeds (*G. gallus domesticus*) had more ultra-long telomeres than the ancestral red jungle fowl (*G. gallus gallus*).	positive	three strains	captive	yes	comparing strains/species	[40]
mouse (*Mus* spp.)	TL was shorter in wild *M. spretus* and *M. caroli* than in both inbred and wild-caught *M. musculus*.	positive (none)	up to eight strains incl. three lab strains.	captive	yes	comparing strains/species	[41]
mouse (*Mus musculus* and *M. spretus*)	TLs from 12 *M. musculus* strains were all >30 kb, while TLs of nine wild-derived *M. spretus* strains were <25 kb (data not shown).	positive	21 strains	captive	yes	comparing strains/species	[42]
mouse (*Mus* spp.)	TL was shorter in wild-derived *M. spretus* than in inbred *M. musculus*. Hybrids had a bimodal distribution of TLs.	positive	two strains	captive	yes	comparing strains/species	[43]
house mouse (*Mus musculus*)	recently inbred mouse strains had shorter TL than established inbred mouse strains.	positive	up to eight species incl. four lab strains	captive	yes	comparing strains/species	[23]
house mouse (*Mus musculus*)	TL was longer in inbred laboratory strains of mice compared with a wild-caught mouse.	positive	individuals from two strains	captive	yes	comparing strains/species	[44]
house mouse (*Mus musculus*)	TL was longer in an inbred laboratory strain compared with a wild-derived strain (*M. m. castaneus*). However, telomerase activity of the two strains was not different.	positive	two strains	captive	yes	comparing strains/species	[45]
mouse (*Mus* spp. and *Peromyscus* spp.)	inbred lines of *P. leucopus*, *M. musculus* and *M. spretus* had longer TLs than outbred lines. Hybrid TL was intermediate to parental TLs.	positive	six strains incl. three lab strains (90 individuals)	captive	yes	comparing strains/species	[24]*
white-footed mouse (*Peromyscus leucopus*)	inbred stock had longer telomeres than an outbred stock. Wild-caught mice had TLs in between inbred and outbred.	positive	157 individuals across five strains	captive	yes	comparing strains/species	[46]*
Norway rat (*Rattus norvegicus*)	TL was shorter in one laboratory rat compared with one wild-caught rat.	negative	two individuals from two strains	captive	yes	comparing strains/species	[44]
Norway rat (*Rattus norvegicus*)	TL was longer in outbred Sprague Dawley rats compared with inbred brown Norway rats.	negative	12–20 individuals	captive	yes	comparing strains/species	[47]
Lagomorphs (Lagomorpha)	European rabbit New Zealand White laboratory strain of *Oryctolagus cuniculus* appeared to have longer TL than jack rabbit, swamp rabbit and pika.	positive	four species	captive	yes	comparing strains/species	[22]
mammals (Mammalia)	domesticated species have longer telomeres in a model accounting for differences in telomerase activity, body mass and lifespan across species.	positive	57 species incl. nine domesticated	captive^2^	yes	comparing strains/species	[25]*
B (15 studies)							
white starfish (*Coscinasterias tenuispina*)	populations with lower genotype diversity (higher clonality) had longer telomeres.	positive	58 individuals from four populations	wild	no	comparing population genotype diversity estimated from 12 microsatellites	[48]
natterjack toads (*Epidalea calamita*)	there were no significant associations between inbreeding coefficient and TL (*n* = 211) or the change in TL (*n* = 25).	none	up to 211 individuals	wild	no	inbreeding coefficients estimated from 16 microsatellites	[27]*
sand lizard (*Lacerta agilis*)	TL was positively associated with hatchlings' level of heterozygosity (*n* = 1658, *p* < 0.0002), but not when controlling for parental identities (*n* = 1340, *p* > 0.854).	none (negative)	up to 1658 individuals	wild	no	heterozygosity estimated from 21 microsatellites	[16]*
white-throated dipper (*Cinclus cinclus*)	no association between TL and inbreeding (*n* = 177, *p* = 0.14), but a non-significant positive tendency when including residual maternal TL (*n* = 114, *p* = 0.05).	none	up to 177 individuals	wild	no	inbreeding coefficients estimated from pedigree based on social parents	[49]*
Seychelles warblers (*Acrocephalus sechellensis*)	TL was negatively associated with homozygosity in juvenile and adult wild birds. Higher maternal homozygosity predicted shorter offspring TL.	negative	up to 592 individuals	wild	no	homozygosity estimated from 30 microsatellites	[19]*
house sparrow (*Passer domesticus*)	negative association between TL and genomic inbreeding estimates (*n* = 371). Non-significant negative association between TL and pedigree-based inbreeding estimates (*n* = 2662).	negative	up to 2662 individuals from two populations	wild	no	genome-wide SNP- and microsatellite-based inbreeding coefficients	[17]*, [50]*
Western burrowing owls (*Athene cunicularia hypugaea*)	negative association between TL and genomic inbreeding estimates.	negative	27 individuals	wild^3^	no	runs of homozygosity estimated from whole-genome sequencing	[20]*
golden-collared manakin (*Manacus vitellinus*), white-collared manakin (*M. candei*)	hybrids had higher average heterozygosity and longer TL. However, there was a negative association between heterozygosity and TL (and TL attrition) within the low-heterozygosity population, *M. candei*, but no associations within the *M. vitellinus* or the hybrid population.	negative/positive / none	up to 127 individuals	wild	no	genome-wide SNP heterozygosity and a hybrid index	[51]*
human (*Homo sapiens*)	three of four individuals in a consanguineous family had shorter telomeres than age-matched controls, reduced telomerase activity, homozygous TERT mutations and suffered from dyskeratosis congenita.	negative	four individuals	-	-	consanguine marriage	[52]
human (*Homo sapiens*)	no association between TL and inbreeding among patients with bipolar I disorder and controls ( *n*= 222, *p* = 0.604), but a negative association was found within schizophrenic patients and controls (*n* = 120, *p* = 0.010).	none (negative)	Up to 342 individuals	-	-	inbreeding coefficients based on 64 short tandem repeat polymorphisms	[53]*
human (*Homo sapiens*)	an individual of first-degree relative consanguinity had much shorter TL than age percentiles, was homozygous for several telomere genes and suffered from dyskeratosis congenita.	negative	one individual	-	-	genome-wide SNP homozygosity	[54]
human (*Homo sapiens*)	a homozygous RTEL1 variant was identified in an inbred family, which was negatively associated with TL and shortening rate.	negative	272 incl. nine inbred individuals	-	-	consanguine marriage and RTEL1 homozygosity	[55]
human (*Homo sapiens*)	the child of second-degree cousins had shorter telomeres than age-matched controls and family members and suffered from a telomere biology disorder owing to a homozygous variant in the TERT gene.	negative	four individuals	-	-	consanguine marriage	[56]
human (*Homo sapiens*)	an individual of first-cousin parentage with a homozygous variant of PARN had shorter TL than three age-matched controls.	negative	four individuals	-	-	consanguine marriage	[57]
human (*Homo sapiens*)	no difference in TL between offspring of unrelated and first cousin marriages.	none	119 individuals	-	-	consanguine marriage	[58]

^1^*C. elegans* is classified as domesticated with reference to [59] and *N. furzeri* as not domesticated with reference to [60].

^2^This study included domesticated animals in captivity, wild-derived captive zoo animals and wild animals as described in [61], from which the data are derived.

^3^Wild-caught owl juveniles may have spent up to two weeks acclimatizing to captivity before TL measurement. The data include TL measurements from the offspring of owls that overwintered in outdoor aviaries but were released prior to reproduction.

^*^Studies included in the quantitative meta-analyses (figure 1 and figure 2).

Next, we conducted a quantitative meta-analysis of the differences in mean absolute TL between species or strains with presumed different histories of inbreeding owing to domestication or captive breeding (table 1A; supplementary material, table S2). Effect sizes were calculated using Hedges’ *g*, a measure of standardized mean difference between two groups [63], using the heteroscedastic estimator with the function ‘escalc’ in the *metafor* 5.0-1 package [64]. Positive effect sizes indicate longer TL in domesticated groups relative to natural groups. Sampling variances for each effect size were calculated from group-specific sample sizes and standard deviations.

Furthermore, we conducted a quantitative meta-analysis of the effect of individual levels of inbreeding on TL, which effectively limits the data to studies on natural populations including wild animals and humans (table 1B; supplementary material, table S3). Reported effect sizes were extracted as regression coefficients describing the association between individual inbreeding coefficient and TL. Effect directions were harmonized such that negative effect sizes consistently represented shorter telomeres with increasing homozygosity or inbreeding. For studies that reported multiple models estimating the same effect with the same data but controlling for different factors, the estimate was extracted from the model identified by the authors as the best-fitting. However, we also performed a non-aggregated meta-analysis including an estimate ID nested within study ID as a random effect to account for within-study effects. Effect sizes were standardized as correlation-equivalent effect sizes: regression coefficients and corresponding standard errors were first converted to Wald statistics, which were transformed to approximate correlation coefficients following standard conversion methods [65]. Correlation coefficients were then transformed to Fisher's *z_r_* values using the zcor measure in the ‘escalc’ function in *metafor* [64]. Sampling variances were approximated as (1/(*n* – 3)), where *n* was the study sample size. Effect sizes were then synthesized using random-effects meta-analysis implemented in *metafor* using restricted maximum likelihood estimation.

For both quantitative meta-analyses, we obtained pruned phylogenetic trees from the open Tree of Life database using *rotl* 3.1.1 [66]. Using *ape* 5.8-1 [67], branch lengths were computed with the method of Grafen [68] and a phylogenetic correlation matrix was constructed. When sample sizes are very small, phylogenetic variance estimates become unstable and it is advisable to include both non-phylogenetic and phylogenetic analyses as qualitative sensitivity tests [69,70]. As random effects, we fitted study ID to account for study-specific effects and the phylogenetic correlation matrix to account for phylogenetic non-independence. However, because species and study identity were highly confounded (i.e. most species were represented by a single study), we had to separate these variance components. Owing to the small sample sizes, we also included a model with no random effects. We therefore compare four models: a fixed effects model, a standard random effects model, and models including either between-study variance or phylogenetic variance.

We additionally assessed publication bias by visually inspecting contour-enhanced funnel plots and performing Egger's regression (regression of standardized effect sizes against their precision, estimated as the inverse of the standard error [71]) as implemented in *metafor* [64]. All analyses were performed in R v. 4.5.2 [72].

## 2. Opposing signals in direction of effects of inbreeding in natural versus domesticated species

We identified 37 studies including 86 species (number of species of mammals *n* = 64, birds *n* = 7, reptiles *n* = 1, amphibians *n* = 1, fish *n* = 6, echinoderms *n* = 1, nematodes *n* = 1, yeast *n* = 2 and angiosperms *n* = 3) spanning ten major classes of eukaryotes (table 1). A contrasting pattern emerges from this synthesis: when comparing different species or strains with assumed different histories of inbreeding owing to, for example, domestication or captive breeding, 77% (17/22) of studies inferred positive associations between TL and inbreeding levels across populations, while only two studies reported a negative association (tables 1A and 2). However, when comparing TL with measured levels of inbreeding within natural populations including humans, 57% (8/14) of studies found negative associations between TL and inbreeding level while only a single study reported a positive association (tables 1B and 2). One study with ambiguous findings was excluded from the vote counting (discussed in §4). This difference in direction between comparison types (table 1A,B) was statistically significant (odds ratio = 51.0, Fisher's exact test *p* = 0.0001). However, these results should be interpreted cautiously, as they are based on directional classifications and do not account for variation in effect sizes, study design or statistical power.

**Table 2.** Overview of the number of studies comparing telomere lengths across strains and species (table 1A) or across individuals (table 1B), and whether they inferred generally positive, negative or no associations between telomere length and inbreeding levels (based on total datasets, table 1). One study with ambiguous findings [51] is not included in this table. Studies comparing strains or species are generally on domesticated organisms in captive environments, while studies comparing individuals are on humans and wild organisms in natural environments. The difference in the direction of effects between comparison types (table 1A,B) was statistically significant (odds ratio = 51.0, Fisher's exact test *p* = 0.0001).

	Positive	None	Negative	Total
**Comparing strains/species**	17	3	2	22
**Comparing individuals**	1	5	8	14
**Total**	18	8	10	36

The quantitative meta-analyses within each comparison type were limited to only eight species across nine studies (figure 1) and nine species across eight studies (figure 2). When comparing mean TLs across domesticated or captive-bred groups with their natural counterparts across studies, all models supported a positive Hedges’ *g*, suggesting that domesticated groups have longer telomeres within this small data subset (model accounting for study ID: Hedges’ *g* = 2.92, 95% CI = [0.72, 5.12], *p* = 0.009, figure 1). Substantial heterogeneity was present across effect sizes in all analyses (*Q*-test *p *< 0.0001; *I^2^* = 99%). Accounting for phylogenetic non-independence increased uncertainty in the overall effect and substantially worsened model fit (ΔAIC = 22.6 relative to the study ID model). This probably reflects insufficient taxonomic replication (or limited phylogenetic signal), reducing the ability to estimate phylogenetic variance robustly. When comparing correlation-equivalent effect sizes of inbreeding on TL across individuals in natural populations across studies, all models supported a small negative effect with CIs overlapping zero (except for the fixed effects model showing a significant negative effect), suggesting no general significant effect of inbreeding on TL within this small data subset (model accounting for study ID: Fisher's *z_r_* = –0.03, 95% CI = [–0.16, 0.11], *p* = 0.690; figure 2). Considerable heterogeneity was present across effect sizes in all analyses (*Q*-test *p* < 0.0001; *I^2^* = 86%). Again, accounting for phylogenetic non-independence further increased uncertainty in the overall effect and worsened model fit (ΔAIC = 3.75 relative to the study ID model).

**Figure 1.**
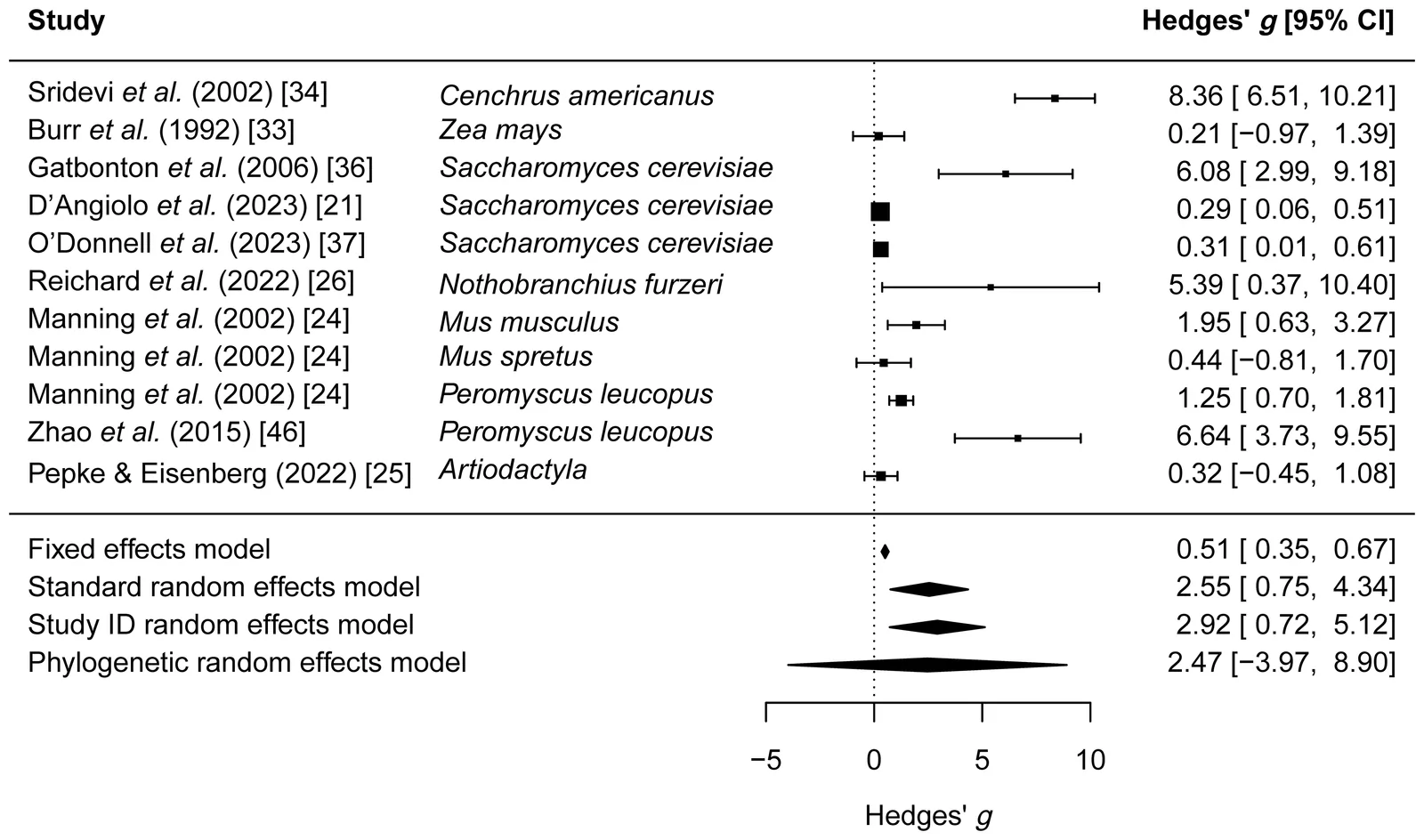
Forest plot of studies included in the meta-analysis comparing absolute telomere lengths across species or strains with assumed different histories of inbreeding owing to domestication or captive breeding against their natural or outbred counterparts (table 1A; supplementary material, table S2). The estimate marks are scaled by sample size and show Hedges’ *g* and corresponding 95% confidence intervals (CI). Below are the pooled estimates of models with no random effects, or with standard, study identity or phylogenetic random effects. All models supported a positive Hedges’ *g*, suggesting that domesticated groups have longer telomeres within this small data subset, but accounting for phylogenetic non-independence greatly increased uncertainty in the overall effect.

**Figure 2.**
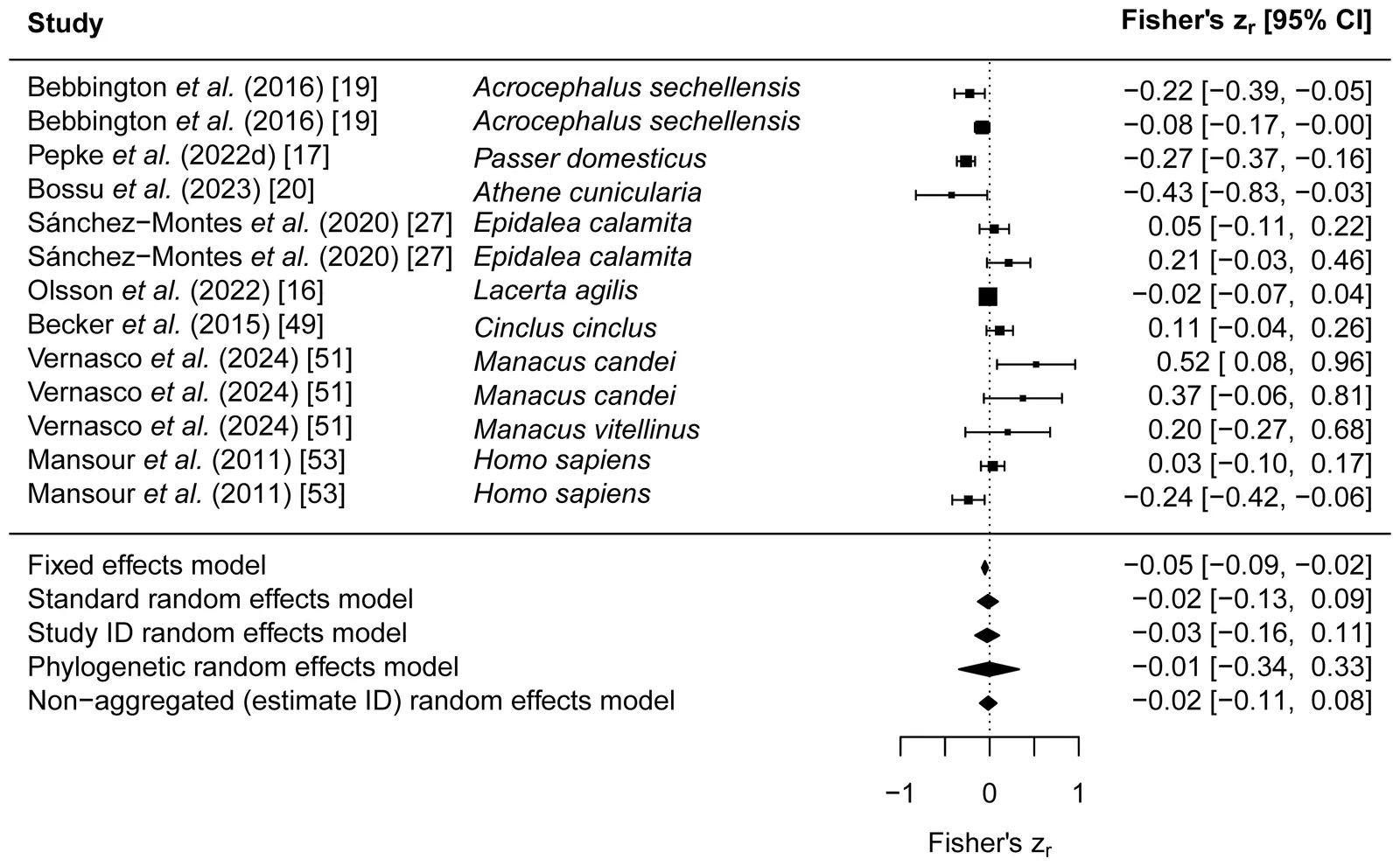
Forest plot of studies included in the meta-analysis comparing effect sizes of inbreeding on TL across individuals in natural populations across studies (table 1B; supplementary material, table S3). The estimate marks are scaled by sample size and show Fisher's *z_r_* and corresponding 95% confidence intervals (CI). Below are the pooled estimates of models with no random effects, or with standard, study identity, phylogenetic or estimate identity random effects. All models supported a small negative effect of similar magnitude with CIs overlapping zero (except for the fixed effects model), suggesting no significant negative effect of inbreeding on telomere length within this small data subset.

Within the studies on domesticated and captive-bred organisms (figure 1), we detected significant asymmetry in the funnel plot (*z* = 3.40, *p* = 0.001), meaning that smaller or less precise studies tended to report more extreme effect sizes. However, interpretation should remain cautious because the dataset contains relatively few studies with consistently positive effect directions and substantial heterogeneity across studies and taxa. Under such conditions, funnel plot asymmetry may reflect between-study heterogeneity or methodological differences rather than publication bias alone [71]. Within the studies on natural populations (figure 2), we detected weak evidence for asymmetry (*z* = 1.67, *p* = 0.100), but the low number of studies and generally small effect sizes limit the statistical power to detect asymmetry reliably [71].

## 3. Domestication can elongate telomeres in captive populations

Domestication is typically associated with demographic bottlenecks and reduced effective population sizes, which can lead to decreased genetic diversity and increased homozygosity relative to wild populations [28,73]. None of the studies on domesticated species (table 1) included individual measures of inbreeding but they instead relied on the assumption that domesticated or laboratory species or strains with a long history of captivity are more inbred than wild or recently wild-derived populations. This is often true (e.g. [74]), but the process of domestication can differ across species, resulting in varying levels of inbreeding, mutational and genetic load [28,73]. Irrespective of the underlying association between domestication and inbreeding, we find longer telomeres in populations with a long history of domestication and captive breeding (table 2; figure 1), including several species of mice (Muroidea), lagomorphs (Lagomorpha), chicken (*Gallus gallus*), killifish (*Nothobranchius* spp.), nematodes (*Caenorhabditis elegans*), yeast (*Saccharomyces* spp.) and millet (*Cenchrus* spp.) (and a cross-species comparison of mammalsincluding cow (*Bos taurus*), sheep (*Ovis aries*), pig (*Sus scrofa*), camel (*Camelus dromedarius*), horse (*Equus caballus*), dog (*Canis familiaris*), rat (*Rattus norvegicus*), mouse (*Mus musculus*) and rabbit (*Oryctolagus cuniculus*); table 1A). This provides support for the hypothesis that the longer telomeres of laboratory strains and domesticated species are not owing to inbreeding *per se* (e.g. [24]), but owing to other effects of captive breeding or domestication (e.g. [23]). These could involve a combination of artificial selection for a faster pace of life with early reproduction, faster growth and decreased energetic constraints in laboratory animals or reduced stressful environmental conditions [25,75–77].

Using an elegant study system, D'Angiolo *et al.* [21] first showed that telomeres in domesticated *Saccharomyces cerevisiae* yeast strains were longer than in wild isolates. Next, in line with our main findings, domesticated *S. cerevisiae* had longer TL than its closest related species, *S. paradoxus*, which was never domesticated. Curiously, isolates derived from fermentation-related processes had longer TL than feral strains that had gone through domestication but then redispersed back into a natural environment [21]. This suggests that telomere elongation during domestication is reversible. D'Angiolo *et al.* [21] also identified genetic variants diverging between domestic and wild strains, which pointed to differences in mitochondrial metabolism underlying differences in TL.

Only two studies on domesticated animals found the opposite pattern, i.e. of shorter telomeres in an inbred compared with an outbred strain, and both studies were on Norway rats (*Rattus norwegicus domesticus*): Seluanov *et al.* [44] reported that lung and skin TLs were shorter in a laboratory rat compared with a single wild-caught rat (*n* = 2), and Fice & Robaire [47] found longer sperm TLs in outbred Sprague Dawley rats compared with inbred brown Norway rats (*n* = 12–20; both strains were transgenic for fluorescent proteins). None of the studies measured inbreeding or provided significance testing for TL differences across strains. However, these observations suggest that influences of captive breeding, inbreeding and domestication on telomere dynamics in rats may be different from other domesticated species, and that these effects may be consistent across somatic and germline tissues. Understanding how telomere dynamics are altered in captive laboratory populations is important for inferring drivers of telomere dynamics and to enable generalizations of model organisms to natural populations.

### (a) Model animals have ultra-long telomeres

Soon after the discovery of the structure and function of telomeres [6], differences in TLs across populations, individuals, cells and chromosomes caught the interest of the research community: Kipling & Cooke [78] reported that telomeres were ultra-long and hypervariable in laboratory mice (*Mus musculus*) compared with humans. Starling *et al.* [41] showed that TL was shorter in wild interfertile *M. spretus* and *M. caroli* than in both inbred and wild-caught *M. musculus*. With these *Mus* species being evolutionarily distinct, Hemann & Greider [23] compared recently inbred *M. musculus* strains (wild-derived 20–30 years ago) with established inbred mouse strains (wild-derived more than 60 years ago). They also found that recently inbred mice had much shorter and less variable TLs than established strains of inbred lab mice. These and other studies in lab mice suggest that TL increases with the duration of the domestication process [23,24,42,43,79].

Cytogenetic work in domesticated chickens (*Gallus gallus domesticus*) has revealed ultra-long telomeres reaching millions of base pairs [80]. These long telomeres are very heterogeneous and occur inconsistently across individuals and cells [40,81]. However, they have been associated with the gene-dense microchromosomes that occur in most birds [40]. Although ultra-long telomeres have been documented in other bird species both in captivity and in the wild [80], we speculate that domestication in captivity could have specific effects on a few loci with ultra-long telomeres rather than affecting TL across all chromosomes. There is some evidence of positive covariation between average TL and average chromosome length [82,83], presumably pertaining primarily to the dynamics of normal macrochromosomes and normal TLs, indicating that different molecular processes may occur at different telomere size ranges.

The observations of long telomeres in lab mice led to concerns about their biological and translational relevance in telomere research [77], and there are still examples of the long telomeres and high telomere shortening rates of lab mice driving associations in comparative studies [70]. Consequently, recent comparative analyses of telomere dynamics have opted to exclude domesticated species [83,84], but without distinguishing captive and wild populations explicitly. Importantly, the long telomeres of lab mice are also observed in other important model organisms, including chicken, nematodes [38] and yeast [21], which questions the generalizability of their telomere dynamics to other systems.

### (b) The variation of animals and plants under domestication

Domesticated organisms share some phenotypic traits that distinguish them from their wild ancestors [1,85], but the existence of a ‘domestication syndrome’ remains contentious [86]. There is, for example, no single directional trend in body size in domesticated compared with wild animal populations [86,87]. However, domesticated species have undergone similar changes that may mirror a faster pace of life [88] in reproductive output and lifespan, fertility, growth rate, genetic diversity, parasite exposure, gut microbiomes, behaviour and energetic constraints [85,86,89], which are factors known to influence variation in TL (e.g. [7,90–94]). Within populations, a faster pace of life has been associated with increased telomere shortening and shorter telomeres [95,96]. However, across species, a faster pace of life has been associated with both increased telomere shortening and longer telomeres [61,83,97,98].

Gleeson & Wilson [99] argued that domestication can disrupt natural reproductive regimes by altering sexual selection, resource availability and maternal stress. Domesticated animals are often selected for elevated reproductive output, which we speculate could increase susceptibility to oxidative damage during reproduction compared with non-domesticated animals [100]. There is no clear evidence for such effects of domestication [100]; however, this could select for longer telomeres [94]. For example, Japanese quails (*Coturnix japonica*) artificially selected for higher maternal investment had longer telomeres early in life, but greater telomere shortening during reproduction in later life [101].

Telomere length is thought to be energetically constrained because telomere maintenance, cell proliferation and replacement are energetically costly [5,76] or because TL serves a signalling function within energetically costly metabolic pathways [102]. Energetic constraints underlie fundamental life-history trade-offs [103], but in captivity, organisms may face decreased energetic demands and reduced environmental variability owing to, e.g. *ad libitum* feeding, reduced costs of feeding and constant temperatures at thermoneutral zones compared with natural environments [75,76]. There is some meta-analytical evidence across vertebrates for a reduced negative response in TL to anthropogenic stressors [104] and higher TL heritability estimates [105] in captivity compared with field studies. Another example is that supplementary feeding of edible dormice (*Glis glis*) in the wild resulted not only in TL maintenance, but in individual telomere elongation during the breeding season [106]. As caloric restriction is a well-known method to increase lifespan [107] and perhaps TL [108] in animals, this may seem counterintuitive. However, caloric restriction studies are carried out in captive environments, and this demonstrates the contrasting energetic schemes of free-living and captive or domesticated animals: it is normal for wild animals to experience the energetic demands and oxidative stress associated with, e.g. foraging effort or food shortages, while captive animals can be decoupled from such trade-offs and benefit from reduced caloric intake.

Domestication or adaptation to captivity may, however, entail costs including elevated cancer rates [109]. Within populations, telomeres that are both too short and too long have been associated with increased cancer risk [76,110]. However, the evolution of short telomeres in larger and longer-lived species (i.e. with a slow pace of life) is generally thought to be a cancer suppression mechanism [25,44,110]. The relationship between TL, cancer risk and domestication remains unexplored, but the potential for novel anticancer adaptations in domesticated species [109] in light of inbreeding [111] and elongated telomeres is an important avenue for future research.

Alternatively, long telomeres could have been ancestral in domesticated species and involved in the early stages of domestication or adaptation to captivity. For example, in chicken, ultra-long telomeres (albeit fewer) also occur in the ancestral red jungle fowl, suggesting that their existence (but not their prevalence) predates domestication events [40]. An analogy to the initial stages of domestication may be found in fast-growing growth hormone transgenic coho salmon (*Oncorhynchus kisutch* [112]). This growth hormone transgene boosts the size of wild (slow-growing) but not of domesticated (fast-growing) rainbow trout (*Oncorhynchus mykiss* [113]), suggesting that analogous changes have occurred during fish domestication. The transgenic salmon experienced increased telomere shortening, probably owing to the increased growth, but surprisingly, they had longer telomeres than wild-types—despite the greater rate of telomere shortening [112]. These transgenic fish contain a single copy of the inserted growth hormone transgene but otherwise possess the same wild-type genetic background—they are therefore presumably not more inbred than the wild-types. This transgenic salmon strain also had higher levels of antioxidants, oxidative stress parameters and more oxidative damage to proteins compared with wild-types [114]. Pauliny *et al.* [112] suggested that founder effects might explain the longer telomeres of the transgenic strain. However, founder effects are unlikely to explain the consistently elongated telomeres of domesticated organisms found in this study.

## 4. Effects of inbreeding on telomere length in natural animal populations

In natural populations, where individuals develop under ecological constraints and are exposed to fluctuating environmental conditions, inbreeding is expected to manifest through reduced somatic maintenance and increased physiological stress [2]. Inbreeding can lead to expression of deleterious recessive alleles and loss of heterozygosity at loci with heterozygote advantage affecting pathways involved in oxidative balance, DNA repair and cellular turnover [11–13]—processes that are central to telomere maintenance [115,116].

Consistent with this, three studies reported shorter telomeres in more inbred individuals within wild bird populations, including Seychelles warblers (*Acrocephalus sechellensis* [19]), house sparrows (*Passer domesticus* [17]) and Western burrowing owls (*Athene cunicularia hypugaea* [20]). The literature search identified other studies that inferred negative effects of inbreeding on TL in ectotherms, but without directly measuring inbreeding (not included in table 1B). For example, Mira-Jover *et al.* [117] speculated that inbreeding could explain a negative association between shell anomalies (which can be correlated with inbreeding, but also with environmental effects [118]) and TL in spur-thighed tortoises (*Testudo graeca*). Sadler *et al.* [18] speculated that inbreeding could explain shorter telomeres in captive lines of zebrafish (*Danio rerio*) undergoing body size selection compared with random harvesting, assuming considerable size heritability. Three studies found no significant associations between inbreeding and TL in wild animal populations, including white-throated dippers (*Cinclus cinclus* [49]), natterjack toads (*Epidalea calamita* [27]) and sand lizards (*Lacerta agilis* [16]), and two studies found no significant associations within humans [53,58].

Although telomere research is normally dominated by human studies, only one study addressed the association between genome-wide inbreeding levels and TL in humans [53]. There was no general association between TL and inbreeding, but a negative association was found within schizophrenic patients in this study (figure 2; supplementary material, table S3). Other human case studies reporting one or a few homozygous mutations associated with short telomeres and also often with rare telomere diseases in offspring of consanguineous marriages [52,54–57] are included in table 1B and point to the genetic basis underlying inbreeding depression in TL (discussed in §5).

Vernasco *et al.* [51] investigated telomere dynamics in a natural hybrid zone in manakins (*Manacus* spp.). Here, hybrids had higher average genome-wide heterozygosity, a greater range in individual heterozygosities and longer average TL compared with either parental population, suggesting a heterosis effect on TL following genetic admixture. Other studies have similarly suggested heterosis effects (i.e. increased TL in hybrid or outbred offspring) in cattle [119] and birds [17], while this was not observed in mice [24], plants [33,35] or yeast [36,120]. However, within the parental manakin population with the lowest heterozygosity, there was a negative association between heterozygosity and TL and TL attrition, and there was no overlap in heterozygosity between the two parental populations [51]. There were no significant associations within the other populations. The authors suggested that these apparently discrepant results may be explained by the species’ unusual lek mating systems, in which high heterozygosity individuals investing more in metabolically demanding courtship display (males) and reproductive effort (females) have shorter telomeres [121].

Only one other study that measured inbreeding (estimated as microsatellite-based genotype diversity) inferred a significant positive association between population-level TL and inbreeding in a wild organism (white starfish, *Coscinasterias tenuispina* [48]). However, this study compared average genotype diversities and TLs across populations, not individuals, and could therefore not be included in the quantitative meta-analysis. As the starfish are clonal, population genotype diversity mainly reflects the relative ratio of fission (asexual reproduction) versus sexual reproduction. Populations with lower genotype diversity and therefore higher fission rates (clonality) had longer telomeres [48]. This population difference in TLs therefore probably reflects differences in reproductive costs of asexual and sexual reproduction. For example, studies on laboratory planarian flatworms (*Schmidtea mediterranea*) have shown that asexual animals maintain TL somatically by telomerase activity during reproduction by fission, whereas sexual animals do not [122].

These different studies exemplify the heterogeneous effects demonstrated by the small overall effect sizes in figure 2. Individual differences in resource acquisition and allocation [93,123] may mask the physiological consequences of inbreeding on TL in natural populations. Furthermore, some wild animal populations may be inbred but do not (yet) show physiological signs of inbreeding depression [2]. Different measures of inbreeding capture different processes but also vary in precision [124]. For example, genomic estimates of inbreeding (weighted average homozygosity over all loci and the proportion of the genome within runs of homozygosity) revealed strong associations with TL, but these were not significant when using microsatellite pedigree-based estimates of inbreeding in a house sparrow metapopulation [17].

## 5. Genetic architecture and developmental telomere length regulation

Telomeres are inherited from parental gametes, and TL is largely established early in life, where embryonic and juvenile development can shape telomere trajectories (e.g. [125–127]). Understanding the developmental origins of telomere dynamics is therefore important for how genetic architecture ultimately can shape ageing trajectories. Studies on humans (e.g. [128–130]), domestic cattle (*Bos taurus* [131,132]), wild house sparrows [50], laboratory nematodes (*Caenorhabditis elegans* [38]) and yeast (S. *cerevisiae* [21,36]) have shown that TL is a highly polygenic trait: hundreds of genetic loci associated with TL have been identified both within coding and non-coding regions, in addition to epigenetic loci [133]. Many homozygous mutations in telomere maintenance genes are associated with short telomeres [134–136]. Such studies of single loci in the absence of variation in inbreeding levels are not included in table 1 but point to the polygenic basis of how increased homozygosity could lead to shorter telomeres.

Inbreeding can expose the phenotypes of deleterious recessive alleles to allow purging selection [137]. However, the combined effects of dominance and the different genetic contributions of multiple loci can make it difficult to predict the exact impact of inbreeding on a specific polygenic trait. If polygenic, small-effect recessive effects distributed throughout the genome are underlying inbreeding depression in TL, this polygenic load is harder to purge in the wild [138]. We therefore speculate that ancestral inbreeding during the early stages of domestication allowed efficient purging of deleterious mutations but that more recent breeding management could somehow lead to a recovery of TL (e.g. [73,139]). However, it is not clear whether such differences between deep (ancestral) inbreeding associated with domestication bottlenecks and shallow (recent) inbreeding associated with breed formation [73,140,141] could explain the telomere elongation observed across domesticated species.

Domestication may have reshaped the genetic basis of TL [21]. For example, Tet (ten-eleven translocation) proteins maintain telomere stability and length, particularly in embryonic stem cells, by promoting DNA demethylation at subtelomeric regions [142]. Curiously, knock-out of TET1 in an outbred mouse strain (with short telomeres) caused full embryonic lethality but only partial lethality in an inbred strain (with long telomeres [45,143]). This suggests lineage-specific functions of telomere maintenance genes in a domesticated model animal [24]. Furthermore, telomere traits may not be evolving independently but instead may change as a correlated response to selection on life-history traits. Domestication often selects for faster growth and developmental programmes, including earlier maturity and higher fecundity (discussed in §3b). Studies of different species have suggested that TL could be genetically correlated with growth traits [144–146] and lifespan [25,147,148], enabling correlated responses in TL to artificial selection in domesticated animals. For example, in domestic dogs (*Canis familiaris*), TL is positively correlated with breed lifespan (or negatively correlated with breed body size [90]), suggesting that domestication selection had pleiotropic effects (or shaped common environmental effects) on TL and key life-history traits. Disentangling the genetic architecture of TL is complicated by context-dependent environmental effects on telomere dynamics during development: for instance, introducing Mexican spadefoots (*Spea multiplicata*) to a novel (carnivorous) diet revealed a negative genetic correlation between TL and growth that was hidden under natural diet (detritivorous) conditions [146]. Again, this suggests that analogous effects of domestication affecting growth or diet (discussed in §3b) could fundamentally restructure the life-history trade-offs underlying telomere dynamics.

## 6. Telomere dynamics in conservation genomics of endangered populations

Understanding the physiological impacts of inbreeding in endangered populations is important for informing conservation actions including prioritization, genetic rescue, managed breeding and habitat connectivity [2]. However, fitness is hard to measure in the wild, and thus quantifying inbreeding depression is a difficult task [149]. Telomere dynamics have been proposed as a proxy for different fitness components [10,94], and the cumulative influences of various genetic and environmental stressors embody the conservation challenges faced by many species [104]—including inbreeding depression as outlined here.

There is evidence of shorter telomeres in small, declining populations of lizards and birds [150–153] and in bird populations that are maladapted to climatic conditions [154,155]. Whether population-level changes in TL can have demographic consequences [156] and predict population persistence [157] remains unanswered at larger spatiotemporal scales [158]. Furthermore, any practical application of telomere dynamics in conservation genomics also faces important challenges and limitations, including high heterogeneity across individuals, tissues and life stages, and diverse influences of environmental conditions on TL [159]. Yet, the potential negative influence of inbreeding on telomere dynamics should be considered in future studies and systems. For example, the accumulation of short telomeres and telomere damage across generations could anticipate the onset of extinction vortices [151], potentially amplifying effects of inbreeding. Under such a theoretical model, telomere shortening could be both the cause and the consequence of population decline. At the opposite extreme, longer telomeres have been associated with recent range expansion [160], maintenance of dispersal [17], balanced harvesting [18] and hybridization [51].

We note that most of the long-term population studies that have found significant negative effects of inbreeding on TL probably have founder effects, because they were founded by a few individuals, for example on islands [17,19], or because they are endangered populations that have undergone recent drastic changes in population size [19,20]. This means that such populations can have unusual population dynamics, and that inbreeding depression can be common and extreme, increasing the power to detect inbreeding depression. By contrast, studies on widespread species in mainland environments found no significant associations between TL and inbreeding ([27,49]; and to some extent [16]). However, the number of such field studies is still limited, and it is not clear what causes the large heterogeneity in effects of inbreeding on TL across studies. As inbreeding and inbreeding tolerance vary across species and with life-history traits [2,14], it remains important to test such relationships across different populations and environments to establish whether telomere dynamics can be used as biomarkers linking genomic erosion to organismal condition and demographic performance.

## 7. Future directions and limitations for studies on inbreeding and domestication telomere dynamics

There is a surprising lack of well-powered human studies on associations between telomere dynamics and inbreeding, given that such data can be made available from existing population-wide cohort telomere studies [128,130]. Future studies using whole-genome sequencing could simultaneously estimate inbreeding (e.g. [124]) and TL (e.g. [92]) to test whether inbreeding shortens telomeres in the relatively outbred human population, or if domestication-like effects of (agri-)culture [85] have altered our telomere biology. Such studies may also test the persistence of inbreeding effects by including variation in individual telomere shortening rates [27,51] and telomerase activity levels [45,52], which remain understudied.

Some model organisms may have odd telomere dynamics, including mice, chicken, nematodes and yeast (but apparently not rats), which is important to consider when designing experiments to disentangle general influences of inbreeding, hybridization, domestication and captivity on telomere dynamics. Comparative telomere studies should not uncritically mix data derived from domesticated and wild species and should treat data from captive populations (including zoos) cautiously until more is known about the drivers of telomere elongation in captive, domesticated species. Several domesticated species and laboratory organisms are derived from human commensal populations, and there may be unexploited analogies between urbanization, captivity and domestication in telomere dynamics. However, despite their abnormally long telomeres, domesticated laboratory animals remain valuable models in telomere research because their telomere dynamics are often found to be influenced by the same genetic and environmental factors as in non-domesticated organisms and humans.

Decomposing individual variation in TL into various genetic and environmental sources through a type of mixed-effect ‘animal’ model using all relationships in a pedigree has become a popular tool in telomere ecology [105], and some of the telomere–inbreeding associations in this review are derived from such studies (table 1B). However, these models rarely include individual inbreeding coefficients as a covariate, even though analyses that do not include this component can return biased estimates of additive genetic variances [161] in the presence of inbreeding depression in TL. Future studies disentangling genetic and environmental components of variation in TL in animal populations using pedigrees should avoid confounding estimates of, e.g. heritabilities by not conditioning models on inbreeding coefficients, which would simultaneously reveal associations between inbreeding and TL. Furthermore, several studies have shown that failing to control for parental effects may inflate effects of inbreeding on TL (in multi- and iteroparous species, e.g. [16,49]), highlighting the importance of careful model construction and data collection for disentangling the true relationship between inbreeding and TL.

There is a lack of studies investigating individual realized inbreeding and telomere dynamics within domesticated species and in comparison with their wild or feral counterparts, and the available studies may be subject to publication bias (§2). During the earliest stages of domestication, domesticated strains may not yet be more inbred than wild strains [140,162]. In some cases, domesticated animals could even have lower levels of inbreeding than their wild counterparts, for example, if the wild populations are small, fragmented or have undergone recent genetic bottlenecks. This is the case in some populations of sheep (*Ovis* spp.) and goats (*Capra* spp. [163]), which emphasizes the need to quantify inbreeding. Furthermore, TL and inbreeding could well be negatively associated within domesticated populations because inbreeding depression can affect domesticated species in similar negative ways to in wild species [141,164]. However, inbreeding depression is often stronger under environmental stress [14], and the environment could be masking inbreeding effects on TL in captivity [104,165]. Thus, comparing intraspecific variation in TLs of wild, feral and domesticated populations remains a valuable approach to further disentangle the causes of TL changes with captivity or domestication.

## 8. Concluding remarks

The apparent paradox of how inbreeding influences telomere dynamics can be resolved by distinguishing domesticated, captive populations from wild, free-living populations, including humans. We speculate that this may reflect different regimes of selection on somatic maintenance in wild versus captive populations (figure 3). In the wild, limited resources and stronger natural selection on somatic maintenance, combined with inbreeding exposing deleterious alleles, result in shorter telomeres. However, in captivity, relaxed energetic and ecological constraints and artificial selection reshape or decouple life-history trade-offs, leading to altered investment in somatic maintenance and reproduction associated with selection for longer telomeres, even in the presence of elevated homozygosity. The multifarious effects of domestication and captivity on TL remain intertwined and are not well understood, but there is currently no evidence that telomeres can be elongated owing to effects of inbreeding alone.

**Figure 3.**
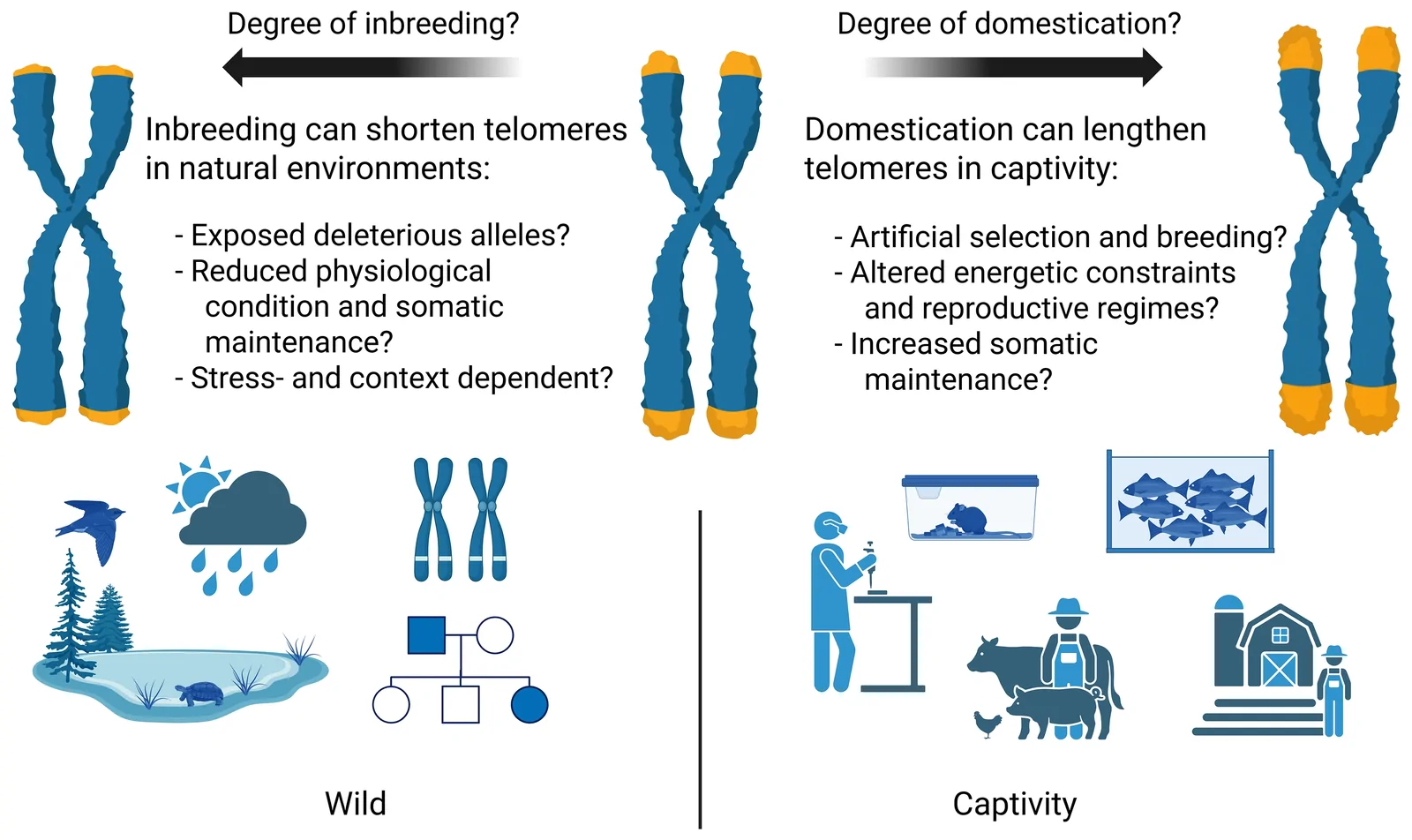
The paradox of how inbreeding influences telomere dynamics may be resolved by distinguishing domesticated populations in captivity from wild, free-living populations in natural environments. This may reflect different regimes of selection on somatic maintenance and energetic constraints in wild versus captive populations. In the wild, inbreeding exposes deleterious alleles resulting in shorter telomeres in homozygous individuals. Domesticated species have longer telomeres than their wild counterparts, but the reasons behind this phenomenon are not well understood. The figure was created using BioRender.com.

There remains substantial unexplained heterogeneity among effect sizes across studies of both domesticated organisms and natural populations, highlighting the need for additional data, including null results. Moreover, within natural populations, only some studies reported negative effects of inbreeding on TL, and the quantitative meta-analysis detected no significant overall effect, emphasizing the context-dependency and generally small magnitude of these relationships. Nevertheless, while telomere research has historically focused primarily on mammals and birds, our synthesis revealed unexpectedly broad taxonomic representation, including endotherms and ectotherms, but also plants and unicellular eukaryotes with distinct telomere maintenance mechanisms. This suggests evolutionarily conserved responses in telomere dynamics to domestication and captivity, opening new lines of enquiry for exploring the underlying mechanisms generating the extraordinary diversity in patterns of life-history strategies and ageing across and within species.

## Supplementary Material



## Data Availability

All data are included in table 1 in the main text and in tables S2 and S3 in the supplementary material [166].

## Theme

One contribution of 21 to a theme issue ‘Developmental circuits in ageing’.
