# Supplementary material for: Disentangling the paradox of inbreeding and telomere dynamics
Source: Philos Trans R Soc Lond B Biol Sci. 2026 Nov 12;381(1963):20260110. doi: 10.1098/rstb.2026.0110 (PMC13755904; doi:10.1098/rstb.2026.0110)
Supplement: Supplementary file 1 [file rstb.2026.0110.zip › rstb.2026.0110/rstb20260110_si_001.docx]

**Supplementary material**

Pepke & Limborg (2026) Disentangling the paradox of inbreeding and telomere dynamics, *Phil. Trans. R. Soc. B.*

**Identification of studies *via* databases**

Studies identified from *Web of Science* (*N* = 392)

Additional studies identified from forward and backward reference searches etc. (*N* = 16)

Duplicates removed (*N* = 2)

**Identification**

Studies not comparing telomere dynamics and inbreeding or domestication levels excluded (*N* = 369)

Conference abstract with no data (*N* = 1)

Retracted (*N* = 1)

Studies screened (*N* = 408)

**Screening**

Studies included in review

(*N* = 37, Table 1)

Studies comparing telomere lengths of species or strains with assumed different histories of inbreeding due to domestication or a long history of captive breeding (*N* = 22, Table 1A)

Studies included in quantitative meta-analysis (*N* = 9, Table S2)

**Included**

Studies comparing telomere lengths with individual levels of inbreeding within natural or wild populations including humans (*N* = 15, Table 1B)

Studies included in quantitative meta-analysis (*N* = 9, Table S3)

**Fig. S1.** PRISMA chart of the literature search process using Web of Science (Clarivate). Forward and backward reference searches were additionally carried out using Web of Science and Google Scholar. Empirical studies were individually screened to assess whether they compared TLs or changes in TLs between individuals or groups with measured or assumed different levels of inbreeding or domestication in eukaryotic organisms.

**Table S1.** This table extends Table 1 in the main text with information on the methods of telomere length (TL) measurement including telomere restriction fragment (TRF), fluorescence in situ hybridization (FISH), quantitative polymerase chain reaction (qPCR), and whole genome sequencing (WGS) methods. Furthermore, the origin of the cells in which TL was measured, is listed.

| Species | TL-inbreeding direction | Captive or wild? | Domesti-cated? | TL origin | TL method | References |
| --- | --- | --- | --- | --- | --- | --- |
| Maize (*Zea mays*) | None | Captive | Yes | Leaves | TRF | Burr *et al.* (1992) |
| Pearl millet (*Pennisetum* spp. [*Cenchrus* spp.]) | Positive | Captive | Yes | Seedlings | TRF | Sridevi *et al.* (2002) |
| Thale cress (*Arabidopsis thaliana*) | None | Captive | Yes | Leaves or flowers | TRF | Maillet *et al.* (2006) |
| Yeast (*Saccharomyces cerevisiae*) | Positive | Captive | Yes | Whole organism | TRF | Gatbonton *et al.* (2006) |
| Yeast (*Saccharomyces cerevisiae* and *S. paradoxus*) | Positive | Captive | Yes | Whole organism | WGS | D’Angiolo *et al.* (2023) |
| Yeast (*Saccharomyces cerevisiae*) | None | Captive | Yes | Whole organism | WGS | O’Donnell *et al.* (2023) |
| Nematode (*Caenorhabditis elegans*) | Positive (none) | Captive | No | Whole organism | WGS | Cook *et al.* (2016) |
| Turquoise killifish (*Nothobranchius* *furzeri*) | Positive | Captive | No | Muscle and skin | TRF, FISH and qPCR | Hartmann *et al.* (2009) |
| Killifish (*Nothobranchius* spp.) | Positive | Captive | No | Muscle (fin) | TRF | Reichard *et al.* (2022) |
| Chicken (*Gallus gallus*) | Positive | Captive | Yes | Blood | TRF and FISH | O’Hare and Delany (2009) |
| Mouse (*Mus* spp.) | Positive (none) | Captive | Yes | Spleen and kidney | TRF | Starling *et al.* (1990) |
| Mouse (*Mus musculus* and *M. spretus*) | Positive | Captive | Yes | Liver | TRF | Prowse and Greider (1995) |
| Mouse (*Mus* spp.) | Positive | Captive | Yes | Spleen | FISH | Zhu *et al.* (1998) |
| House mouse (*Mus musculus*) | Positive | Captive | Yes | Liver and spleen | TRF and FISH | Hemann and Greider (2000) |
| House mouse (*Mus musculus*) | Positive | Captive | Yes | Lung and skin | TRF | Seluanov *et al.* (2008) |
| House mouse (*Mus musculus*) | Positive | Captive | Yes | Muscle | TRF | O'Connor *et al.* (2009) |
| Mouse (*Mus* spp. and *Peromyscus* spp.) | Positive | Captive | Yes | Blood | FISH | Manning *et al.* (2002) |
| White-footed mouse (*Peromyscus* *leucopus*) | Positive | Captive | Yes | Blood | TRF | Zhao *et al.* (2015) |
| Norway rat (*Rattus norvegicus*) | Negative | Captive | Yes | Lung and skin | TRF | Seluanov *et al.* (2008) |
| Norway rat (*Rattus* *norvegicus*) | Negative | Captive | Yes | Sperm | qPCR | Fice and Robaire (2019) |
| Lagomorphs (Lagomorpha) | Positive | Captive | Yes | Skin | TRF | Forsyth *et al.* (2005) |
| Mammals (Mammalia) | Positive | Captive | Yes | Skin, kidney, lung or cornea | TRF | Pepke and Eisenberg (2022) |
| White starfish (*Coscinasterias tenuispina*) | Positive | Wild | No | Muscle (tube feet) | qPCR | Garcia-Cisneros *et al.* (2015) |
| Natterjack toads (*Epidalea* *calamita*) | None | Wild | No | Toe tip (incl. muscle, skin, cartilage, bone) | qPCR | Sánchez-Montes *et al.* (2020) |
| Sand lizard (*Lacerta agilis*) | None (negative) | Wild | No | Blood and tissue | qPCR | Olsson *et al.* (2022) |
| White-throated dipper (*Cinclus cinclus*) | None | Wild | No | Blood | qPCR | Becker *et al.* (2015) |
| Seychelles warblers (*Acrocephalus* *sechellensis*) | Negative | Wild | No | Blood | qPCR | Bebbington *et al.* (2016) |
| House sparrow (*Passer domesticus*) | Negative | Wild | No | Blood | qPCR | Pepke *et al.* (2022b); Pepke *et al.* (2022a) |
| Western burrowing owls (*Athene cunicularia hypugaea*) | Negative | Wild | No | Blood | qPCR | Bossu *et al.* (2023) |
| Golden-collared manakin (*Manacus vitellinus*), White-collared manakin (*M. candei*) | Negative / positive / none | Wild | No | Blood | qPCR | Vernasco *et al.* (2024) |
| Human (*Homo sapiens*) | Negative | - | - | Blood | qPCR and TRF | Marrone *et al.* (2007) |
| Human (*Homo sapiens*) | None / negative | - | - | Blood | qPCR | Mansour *et al.* (2011) |
| Human (*Homo sapiens*) | Negative | - | - | Blood | FISH | Ungar *et al.* (2018) |
| Human (*Homo sapiens*) | Negative | - | - | Blood | TRF | Gutierrez-Rodrigues *et al.* (2019) |
| Human (*Homo sapiens*) | Negative | - | - | Blood | qPCR | Çepni *et al.* (2022) |
| Human (*Homo sapiens*) | Negative | - | - | Not reported | qPCR | Cheng *et al.* (2024) |
| Human (*Homo sapiens*) | None | - | - | Blood | qPCR | Darvishi and Saadat (2025) |

**Table S2.** Data from studies from Table 1A used in the meta-analysis of mean absolute telomere length (kbp) in inbred groups (mi) and the associated standard deviation (sdi) and sample size (ni) and mean absolute telomere length in natural groups (mn) and the associated standard deviation (sdn) and sample size (nn).

| Reference | Study  ID | mi | sdi | ni | mn | sdn | nn | Species | Comment |
| --- | --- | --- | --- | --- | --- | --- | --- | --- | --- |
| Sridevi et al. (2002) | 1 | 24.65625 | 0.66875 | 32 | 17.63333 | 0.961667 | 24 | *Cenchrus americanus* | Synonymous with *Pennisetum glaucum*. |
| Burr et al. (1992) | 2 | 6.15 | 3.461936 | 8 | 5.525 | 1.744097 | 4 | *Zea mays* |  |
| Gatbonton et al. (2006) | 3 | 0.35 | 0.009899 | 2 | 0.256 | 0.018028 | 13 | *Saccharomyces cerevisiae* | SD estimated from SE. |
| D’Angiolo et al. (2023) | 4 | 0.241 | 0.111 | 430 | 0.215 | 0.065 | 52 | *Saccharomyces cerevisiae* |  |
| O’Donnell et al. (2023) | 5 | 0.311581 | 0.138639 | 105 | 0.273578 | 0.100759 | 65 | *Saccharomyces cerevisiae* |  |
| Reichard et al. (2022) | 6 | 7.33 | 0.5023 | 3 | 5.215714 | 0.18377 | 20 | *Nothobranchius furzeri* | NF222 strain excluded by the authors due to different  ages at sampling. SD estimated from SE. |
| Manning et al. (2002) | 7 | 54 | 12 | 9 | 35 | 4.898979 | 6 | *Mus musculus* | Estimated from Fig. 3. SD estimated from SE. |
| Manning et al. (2002) | 7 | 36 | 7.348469 | 6 | 33 | 4.898979 | 6 | *Mus spretus* | Estimated from Fig. 3. SD estimated from SE. |
| Manning et al. (2002) | 7 | 73.67 | 39.04 | 38 | 33 | 22.91 | 21 | *Peromyscus leucopus* | Estimated from Fig. 1B. SD estimated from SE. |
| Zhao et al. (2015) | 8 | 20.36 | 2.88 | 9 | 5.39 | 1.232883 | 38 | *Peromyscus leucopus* | Comparing an inbred and an outbred strain.  SD estimated from SE. |
| Pepke & Eisenberg (2022) | 9 | 24.8 | 14.3 | 9 | 20.3 | 13.7 | 48 | *Bos taurus* | This cross-species comparison included mainly domesticated members of Artiodactyla (but also of Perissodactyla, Carnivora, Rodentia and Lagomorpha) compared to non-domesticated members of Mammalia, hence *Bos taurus* was used as an approximate phylogenetic placement. |

**Table S3.** Data from studies from Table 1B used in the meta-analysis of the effect (b) of individual inbreeding coefficients on telomere length and the associated standard error (SE) and sample size (n). The comments detail the data subsets giving rise to the different estimates within each study. Best estimate indicates whether an estimate is included in the main (aggregated) model.

| Reference | Study ID | Estimate ID | Best estimate | b | SE | z | n | Species | Comment |
| --- | --- | --- | --- | --- | --- | --- | --- | --- | --- |
| Bebbington et al. (2016) | 1 | 1 | 1 | -1.24 | 0.47 | -2.638297872 | 137 | *Acrocephalus sechellensis* | Juveniles. |
| Bebbington et al. (2016) | 1 | 2 | 1 | -0.14 | 0.07 | -2 | 568 | *Acrocephalus sechellensis* | Adults. |
| Pepke et al. (2022a) | 2 | 3 | 0 | -0.2067 | 0.1843112 | -1.121472813 | 2662 | *Passer domesticus* | Nestlings, F_PED_, Bayesian animal model. |
| Pepke et al. (2022a) | 2 | 4 | 0 | -0.1796 | 0.0941 | -1.908607864 | 2462 | *Passer domesticus* | Nestlings, F_PED_, frequentist linear mixed-effects model. |
| Pepke et al. (2022d) | 3 | 5 | 0 | -0.169 | 0.101 | -1.673267327 | 1195 | *Passer domesticus* | Nestlings, F_PED_ (2 known parents and ≥2 known grandparents). |
| Pepke et al. (2022d) | 3 | 6 | 1 | -1.517 | 0.293 | -5.177474403 | 371 | *Passer domesticus* | Nestlings, F_GRM_. |
| Pepke et al. (2022d) | 3 | 7 | 0 | -1.148 | 0.512 | -2.2421875 | 371 | *Passer domesticus* | Nestlings, F_ROH_ |
| Pepke et al. (2022d) | 3 | 8 | 0 | -0.205 | 0.198 | -1.035353535 | 320 | *Passer domesticus* | Nestlings, F_PED_ (≥2 known full generations). |
| Bossu et al. (2023) | 4 | 9 | 1 | -1.9366 | 0.847 | -2.286422668 | 27 | *Athene cunicularia* | Juveniles, model accounting for year effects only, not controlling for any parental effects. |
| Sánchez-Montes et al. (2020) | 5 | 10 | 1 | 0.26 | 0.42 | 0.624401914 | 143 | *Epidalea calamita* | Males. |
| Sánchez-Montes et al. (2020) | 5 | 11 | 1 | 1.352 | 0.762 | 1.774278215 | 68 | *Epidalea calamita* | Females. |
| Olsson et al. (2022) | 6 | 12 | 0 | -0.036 | 0.01 | -3.6 | 1658 | *Lacerta agilis* | Juveniles, sign changed hetero- to homozygosity. |
| Olsson et al. (2022) | 6 | 13 | 1 | -0.006 | 0.009 | -0.666666667 | 1348 | *Lacerta agilis* | Juveniles with parental ID, sign changed hetero- to homozygosity. |
| Becker et al. (2015) | 7 | 14 | 1 | 0.53 | 0.35 | 1.514285714 | 177 | *Cinclus cinclus* | All individuals. |
| Becker et al. (2015) | 7 | 15 | 0 | 0.87 | 0.43 | 2.023255814 | 114 | *Cinclus cinclus* | Including maternal TL. |
| Vernasco et al. (2024) | 8 | 16 | 1 | 0.68 | 0.26 | 2.615384615 | 23 | *Manacus candei* | Sign changed hetero- to homozygosity. |
| Vernasco et al. (2024) | 8 | 17 | 1 | 0.11 | 0.06 | 1.833333333 | 23 | *Manacus candei* | Hybrid population of *M. candei* and *M. vitellinus*, sign changed hetero- to homozygosity. |
| Vernasco et al. (2024) | 8 | 18 | 1 | 0.21 | 0.23 | 0.913043478 | 20 | *Manacus vitellinus* | Sign changed hetero- to homozygosity. |
| Mansour et al. (2011) | 9 | 19 | 1 | 0.334 | 0.642 | 0.520249221 | 222 | *Homo sapiens* | Patients with bipolar 1 disorder compared to controls. |
| Mansour et al. (2011) | 9 | 20 | 1 | -1.445 | 0.549 | -2.632058288 | 120 | *Homo sapiens* | Patients with schizophrenia compared to controls. |

**References**

Bebbington, K., Spurgin, L. G., Fairfield, E. A., Dugdale, H. L., Komdeur, J., Burke, T., & Richardson, D. S. (2016). Telomere length reveals cumulative individual and transgenerational inbreeding effects in a passerine bird. *Molecular Ecology*, *25*(12), 2949-2960. <https://doi.org/10.1111/mec.13670>

Becker, P. J. J., Reichert, S., Zahn, S., Hegelbach, J., Massemin, S., Keller, L. F., Postma, E., & Criscuolo, F. (2015). Mother–offspring and nest-mate resemblance but no heritability in early-life telomere length in white-throated dippers. *Proceedings of the Royal Society B: Biological Sciences*, *282*(1807), 20142924. <https://doi.org/10.1098/rspb.2014.2924>

Bossu, C. M., Rodriguez, M., Rayne, C., Chromczak, D. A., Higgins, P. G., Trulio, L. A., & Ruegg, K. C. (2023). Genomic approaches to mitigating genetic diversity loss in declining populations. *Molecular Ecology*, *32*(19), 5228-5240. <https://doi.org/10.1111/mec.17109>

Burr, B., Burr, F. A., Matz, E. C., & Romero-Severson, J. (1992). Pinning down loose ends: mapping telomeres and factors affecting their length. *The Plant Cell*, *4*(8), 953-960. <https://doi.org/10.1105/tpc.4.8.953>

Çepni, E., Satkın, N. B., Moheb, L. A., Rocha, M. E., & Kayserili, H. (2022). Biallelic TERT variant leads to Hoyeraal–Hreidarsson syndrome with additional dyskeratosis congenita findings. *American Journal of Medical Genetics Part A*, *188*(4), 1226-1232. <https://doi.org/10.1002/ajmg.a.62602>

Cheng, H.-C., Nanda, A., Lin, C.-Y., McGrath, J. A., & Hsu, C.-K. (2024). Hoyeraal–Hreidarsson syndrome associated with a new homozygous splice variant in PARN. *Clinical and Experimental Dermatology*, *49*(10), 1284-1287. <https://doi.org/10.1093/ced/llae211>

Cook, D. E., Zdraljevic, S., Tanny, R. E., Seo, B., Riccardi, D. D., Noble, L. M., Rockman, M. V., Alkema, M. J., Braendle, C., Kammenga, J. E., Wang, J., Kruglyak, L., Félix, M.-A., Lee, J., & Andersen, E. C. (2016). The genetic basis of natural variation in *Cenorhabditis elegans* telomere length. *Genetics*, *204*(1), 371-383. <https://doi.org/10.1534/genetics.116.191148>

D’Angiolo, M., Yue, J.-X., De Chiara, M., Barré, B. P., Giraud Panis, M.-J., Gilson, E., & Liti, G. (2023). Telomeres are shorter in wild *Saccharomyces cerevisiae* isolates than in domesticated ones. *Genetics*, *223*(3), iyac186. <https://doi.org/10.1093/genetics/iyac186>

Darvishi, F. Z., & Saadat, M. (2025). Association between leukocytes telomere length and parental consanguineous marriage. *EXCLI Journal*, *24*, 177-178. <https://doi.org/10.17179/excli2024-7920>

Fice, H. E., & Robaire, B. (2019). Telomere dynamics throughout spermatogenesis. *Genes*, *10*(7), 525. <https://doi.org/10.3390/genes10070525>

Forsyth, N. R., Elder, F. F. B., Shay, J. W., & Wright, W. E. (2005). Lagomorphs (rabbits, pikas and hares) do not use telomere-directed replicative aging in vitro. *Mechanisms of Ageing and Development*, *126*(6), 685-691. <https://doi.org/10.1016/j.mad.2005.01.003>

Garcia-Cisneros, A., Pérez-Portela, R., Almroth, B. C., Degerman, S., Palacín, C., & Sköld, H. N. (2015). Long telomeres are associated with clonality in wild populations of the fissiparous starfish *Coscinasterias tenuispina*. *Heredity*, *115*(5), 437-443. <https://doi.org/10.1038/hdy.2015.43>

Gatbonton, T., Imbesi, M., Nelson, M., Akey, J. M., Ruderfer, D. M., Kruglyak, L., Simon, J. A., & Bedalov, A. (2006). Telomere length as a quantitative trait: Genome-wide survey and genetic mapping of telomere length-control genes in yeast. *PLOS Genetics*, *2*(3), e35. <https://doi.org/10.1371/journal.pgen.0020035>

Gutierrez-Rodrigues, F., Masri, N., Chouery, E., Diamond, C., Jalkh, N., Vicente, A., Kajigaya, S., Abillama, F., Bejjani, N., Serhal, W., Calado, R. T., Young, N. S., Farhat, H., & Coussa, M. L. (2019). A novel homozygous RTEL1 variant in a consanguineous Lebanese family: phenotypic heterogeneity and disease anticipation. *Human Genetics*, *138*(11), 1323-1330. <https://doi.org/10.1007/s00439-019-02076-8>

Hartmann, N., Reichwald, K., Lechel, A., Graf, M., Kirschner, J., Dorn, A., Terzibasi, E., Wellner, J., Platzer, M., Rudolph, K. L., Cellerino, A., & Englert, C. (2009). Telomeres shorten while Tert expression increases during ageing of the short-lived fish *Nothobranchius furzeri*. *Mechanisms of Ageing and Development*, *130*(5), 290–296. <https://doi.org/10.1016/j.mad.2009.01.003>

Hemann, M. T., & Greider, C. W. (2000). Wild-derived inbred mouse strains have short telomeres. *Nucleic Acids Research*, *28*(22), 4474–4478. <https://doi.org/10.1093/nar/28.22.4474>

Maillet, G., White, C. I., & Gallego, M. E. (2006). Telomere-length regulation in inter-ecotype crosses of Arabidopsis. *Plant Molecular Biology*, *62*(6), 859-866. <https://doi.org/10.1007/s11103-006-9061-7>

Manning, E. L., Crossland, J., Dewey, M. J., & Van Zant, G. (2002). Influences of inbreeding and genetics on telomere length in mice. *Mammalian Genome*, *13*(5), 234-238. <https://doi.org/10.1007/s003350020027>

Mansour, H., Chowdari, K., Fathi, W., Elassy, M., Ibrahim, I., Wood, J., Bamne, M., Tobar, S., Yassin, A., Salah, H., Elsayed, H., Eissa, A., El-Boraie, H., Ibrahim, N. E., Elsayed, M., El-Bahaei, W., Gomaa, Z., El-Chennawi, F., & Nimgaonkar, V. L. (2011). Does telomere length mediate associations between inbreeding and increased risk for bipolar I disorder and schizophrenia? *Psychiatry Research*, *188*(1), 129-132. <https://doi.org/10.1016/j.psychres.2011.01.010>

Marrone, A., Walne, A., Tamary, H., Masunari, Y., Kirwan, M., Beswick, R., Vulliamy, T., & Dokal, I. (2007). Telomerase reverse-transcriptase homozygous mutations in autosomal recessive dyskeratosis congenita and Hoyeraal-Hreidarsson syndrome. *Blood*, *110*(13), 4198-4205. <https://doi.org/10.1182/blood-2006-12-062851>

O'Connor, M. S., Carlson, M. E., & Conboy, I. M. (2009). Differentiation rather than aging of muscle stem cells abolishes their telomerase activity. *Biotechnology Progress*, *25*(4), 1130-1137. <https://doi.org/10.1002/btpr.223>

O’Donnell, S., Yue, J.-X., Saada, O. A., Agier, N., Caradec, C., Cokelaer, T., De Chiara, M., Delmas, S., Dutreux, F., Fournier, T., Friedrich, A., Kornobis, E., Li, J., Miao, Z., Tattini, L., Schacherer, J., Liti, G., & Fischer, G. (2023). Telomere-to-telomere assemblies of 142 strains characterize the genome structural landscape in *Saccharomyces cerevisiae*. *Nature Genetics*, *55*(8), 1390-1399. <https://doi.org/10.1038/s41588-023-01459-y>

O’Hare, T. H., & Delany, M. E. (2009). Genetic variation exists for telomeric array organization within and among the genomes of normal, immortalized, and transformed chicken systems. *Chromosome Research*, *17*(8), 947-964. <https://doi.org/10.1007/s10577-009-9082-6>

Olsson, M., Bererhi, B., Miller, E., Schwartz, T., Rollings, N., Lindsay, W., & Wapstra, E. (2022). Inbreeding effects on telomeres in hatchling sand lizards (*Lacerta agilis*): An optimal family affair? *Molecular Ecology*, *31*(24), 6605-6616. <https://doi.org/10.1111/mec.16723>

Pepke, M. L., & Eisenberg, D. T. A. (2022). On the comparative biology of mammalian telomeres: Telomere length co-evolves with body mass, lifespan and cancer risk. *Molecular Ecology*, *31*(23), 6286-6296. <https://doi.org/10.1111/mec.15870>

Pepke, M. L., Kvalnes, T., Lundregan, S., Boner, W., Monaghan, P., Sæther, B.-E., Jensen, H., & Ringsby, T. H. (2022a). Genetic architecture and heritability of early-life telomere length in a wild passerine. *Molecular Ecology*, *31*(23), 6360-6381. <https://doi.org/10.1111/mec.16288>

Pepke, M. L., Niskanen, A. K., Kvalnes, T., Boner, W., Sæther, B.-E., Ringsby, T. H., & Jensen, H. (2022b). Inbreeding is associated with shorter early-life telomere length in a wild passerine. *Conservation Genetics*, *23*(3), 639-651. <https://doi.org/10.1007/s10592-022-01441-x>

Prowse, K. R., & Greider, C. W. (1995). Developmental and tissue-specific regulation of mouse telomerase and telomere length. *Proceedings of the National Academy of Sciences*, *92*(11), 4818-4822. <https://doi.org/10.1073/pnas.92.11.4818>

Reichard, M., Giannetti, K., Ferreira, T., Maouche, A., Vrtílek, M., Polačik, M., Blažek, R., & Ferreira, M. G. (2022). Lifespan and telomere length variation across populations of wild-derived African killifish. *Molecular Ecology*, *31*(23), 5979-5992. <https://doi.org/10.1111/mec.16287>

Sánchez-Montes, G., Martínez-Solano, Í., Díaz-Paniagua, C., Vilches, A., Ariño, A. H., & Gomez-Mestre, I. (2020). Telomere attrition with age in a wild amphibian population. *Biology Letters*, *16*(7), 20200168. <https://doi.org/10.1098/rsbl.2020.0168>

Seluanov, A., Hine, C., Bozzella, M., Hall, A., Sasahara, T. H. C., Ribeiro, A. A. C. M., Catania, K. C., Presgraves, D. C., & Gorbunova, V. (2008). Distinct tumor suppressor mechanisms evolve in rodent species that differ in size and lifespan. *Aging Cell*, *7*(6), 813-823. <https://doi.org/10.1111/j.1474-9726.2008.00431.x>

Sridevi, V., Uma, K. D., Sivaramakrishnan, S., & Isola, N. R. (2002). Telomere length as related to chromosome length in the genus *Pennisetum*. *CYTOLOGIA*, *67*(2), 185-190. <https://doi.org/10.1508/cytologia.67.185>

Starling, J. A., Maule, J., Hastie, N. D., & Allshire, R. C. (1990). Extensive telomere repeat arrays in mouse are hypervariable. *Nucleic Acids Research*, *18*(23), 6881-6888. <https://doi.org/10.1093/nar/18.23.6881>

Ungar, R. A., Giri, N., Pao, M., Khincha, P. P., Zhou, W., Alter, B. P., & Savage, S. A. (2018). Complex phenotype of dyskeratosis congenita and mood dysregulation with novel homozygous RTEL1 and TPH1 variants. *American Journal of Medical Genetics Part A*, *176*(6), 1432-1437. <https://doi.org/10.1002/ajmg.a.38706>

Vernasco, B. J., Long, K. M., Braun, M. J., & Brawn, J. D. (2024). Genetic and telomeric variability: Insights from a tropical avian hybrid zone. *Molecular Ecology*, *33*(18), e17491. <https://doi.org/https://doi.org/10.1111/mec.17491>

Zhao, X., Ueda, Y., Kajigaya, S., Alaks, G., Desierto, M. J., Townsley, D. M., Dumitriu, B., Chen, J., Lacy, R. C., & Young, N. S. (2015). Cloning and molecular characterization of telomerase reverse transcriptase (TERT) and telomere length analysis of *Peromyscus leucopus*. *Gene*, *568*(1), 8-18. <https://doi.org/10.1016/j.gene.2015.05.013>

Zhu, L., Hathcock, K. S., Hande, P., Lansdorp, P. M., Seldin, M. F., & Hodes, R. J. (1998). Telomere length regulation in mice is linked to a novel chromosome locus. *Proceedings of the National Academy of Sciences*, *95*(15), 8648-8653. <https://doi.org/10.1073/pnas.95.15.8648>
